# Racial/Ethnic Disparities in Interhospital Transfer for Conditions With a Mortality Benefit to Transfer Among Patients With Medicare

**DOI:** 10.1001/jamanetworkopen.2021.3474

**Published:** 2021-03-26

**Authors:** Evan Michael Shannon, Jie Zheng, E. John Orav, Jeffrey L. Schnipper, Stephanie K. Mueller

**Affiliations:** 1 Department of Medicine, Brigham and Women’s Hospital, Boston, Massachusetts; 2 Harvard Medical School, Boston, Massachusetts; 3 Harvard T.H. Chan School of Public Health, Boston, Massachusetts; 4 Division of General Internal Medicine and Primary Care, Brigham and Women’s Hospital, Boston, Massachusetts

## Abstract

**Importance:**

Interhospital transfer (IHT) of patients is a common occurrence in modern health care. Racial/ethnic disparities are prevalent throughout US health care, but their presence in IHT is not well characterized.

**Objective:**

To determine if there are racial/ethnic disparities in IHT for medical diagnoses for which IHT is associated with a mortality benefit.

**Design, Setting, and Participants:**

This cross-sectional analysis used 2013 data from the Center for Medicare & Medicaid Services 100% Master Beneficiary Summary and Inpatient Claims merged with 2013 American Hospital Association data. Individuals with Medicare aged 65 years or older continuously enrolled in Medicare Part A and B with an inpatient hospitalization claim in 2013 for primary diagnosis of acute myocardial infarction, stroke, sepsis, or respiratory diseases were included. Data analysis occurred from November 2019 through July 2020.

**Exposures:**

Race/ethnicity.

**Main Outcomes and Measures:**

The primary outcome of interest was IHT. For the primary analysis, a series of logistic regression models were created to estimate the adjusted odds of IHT for Black and Hispanic patients compared with White patients, controlling for patient clinical and demographic variables and incorporating hospital fixed effects. In secondary analyses, subgroup analyses were conducted by diagnosis, hospital teaching status, and hospitalization to hospitals in the top decile of Black and Hispanic patient proportion.

**Results:**

Among 899 557 patients, 734 958 patients were White (81.7%), 84 544 patients were Black (9.4%), and 47 588 patients were Hispanic (5.3%); there were 418 683 men (46.5%), and 306 215 patients (34.0%) were older than 84 years. The mean (SD) age was 76.8 (7.5) years. Among all patients, 20 171 White patients (2.7%), 1913 Black patients (2.3%), and 1062 Hispanic patients (2.2%) underwent IHT. After controlling for patient variables and hospital fixed effects, Black patients had a persistently lower odds of IHT (adjusted odds ratio, 0.87; 95% CI, 0.81-0.92; *P* < .001), while Hispanic patients had higher odds of IHT (adjusted odds ratio, 1.14; 95% CI, 1.05-1.24; *P* = .002) compared with White patients.

**Conclusions and Relevance:**

This national evaluation of IHT among patients hospitalized with diagnoses previously found to have mortality benefit with transfer found that, compared with White patients, Black patients had persistently lower adjusted odds of transfer after accounting for patient and hospital characteristics and measured across various hospital settings. Meanwhile, Hispanic patients had higher adjusted odds of transfer. This research highlights the need for the development of strategies to mitigate disparate transfer practices by patient race/ethnicity.

## Key Points

**Question:**

Are there disparities by race/ethnicity in interhospital transfer (IHT) for patients with conditions previously found to have a potential mortality benefit from transfer?

**Findings:**

In this cross-sectional study of 2013 Medicare data merged with American Hospital Association data that included 899 557 patients, after adjusting for patient characteristics and hospital fixed effects, Black patients had significantly lower adjusted odds and Hispanic patients had significantly higher adjusted odds of IHT compared with White patients.

**Meaning:**

There may be differential practices in IHT by race/ethnicity.

## Teaser

This cross-sectional study examines disparities by race/ethnicity in interhospital transfer among among Medicare-enrolled patients with conditions previously found to have a potential mortality benefit from transfer.

## Introduction

Interhospital transfer (IHT) of patients from 1 acute care facility to another is becoming an increasingly common occurrence in modern health care. An estimated 1.5% to 9% of all inpatient admissions to referral hospitals originate from another acute care hospital.^1,2,3^ Typically, IHT is initiated so that patients may receive specialized care unavailable at the origin hospital. There is evidence to suggest that IHT is associated with lower mortality for select conditions.^4,5^

Despite this, there is a great deal of heterogeneity in transfer practices across the United States.^2,6,7,8^ Language surrounding transfer in the Emergency Medical Treatment and Active Labor Act is vague, stating that patients “should” be transferred to a referral hospital if they require procedural care not available at the origin hospital or when “medical benefits outweigh risks,” regardless of payer status, race/ethnicity, sex, income, or other nonclinical factors.^9^ While in some instances there are clear guidelines on when to initiate transfer (eg, revascularization for acute myocardial infarction [AMI] and thrombectomy for proximal large vessel stroke), the decision to transfer is often left to clinicians. Such decisions are prone to influence by nonclinical factors, such as those mentioned above, leading to variability in practice.^2^

There are innumerable instances of health care disparities and inequities by race and ethnicity in the United States across a wide range of metrics.^10,11^ It remains unclear to what extent such disparities exist in IHT. Several large registry-based studies^1,2,3,12,13^ investigating factors associated with IHT have shown that IHT practices may differ based on nonclinical factors, including race/ethnicity. The results from these studies suggest that Black patients^2,3,13^ and Hispanic patients^1,13^ were less likely to undergo IHT, although most of these studies did not specifically investigate the association between race/ethnicity and IHT. Moreover, prior studies^5,7,14,15,16^ investigating disparities in IHT have compared Black patients with White patients, whereas disparities between Hispanic patients and White patients are less well understood. Furthermore, while there is some prior research on IHT disparities in the cardiology literature,^5,7,14,15^ such disparities have not been thoroughly explored among other medical diagnoses with a potential mortality benefit from IHT.^4^

The purpose of this study was to determine if there are racial/ethnicity disparities in IHT practices for diseases for which IHT is associated with a mortality benefit, including AMI, stroke, sepsis, and certain respiratory illness,^4^ using a national sample of patients.

## Methods

This cross-sectional study was approved by the Partners Healthcare Human Subjects Institutional Review Board, which granted a waiver of informed consent on the basis that patients would be exposed to minimal risk from this study. This study followed the Strengthening the Reporting of Observational Studies in Epidemiology (STROBE) reporting guideline for cross-sectional studies.

### Data and Study Population

We performed a cross-sectional analysis of 2013 Center for Medicare & Medicaid Services (CMS) 100% Master Beneficiary Summary and Inpatient claims merged with 2013 American Hospital Association (AHA) data. The CMS claims data set contains demographic and clinical data on all individuals enrolled in fee-for-service Medicare.^17^ The AHA data set includes characteristics for all US acute care hospitals.^18^ Data analysis occurred from November 2019 through July 2020.

Our study group consisted of patients with primary disease categories for which a mortality benefit was previously found to be associated with IHT, namely AMI, stroke, sepsis, and respiratory diseases (eg, mycobacterial infection, fungal infection, aspiration-related lung disease, empyema, lung abscess, and mediastinitis).^4^ We included individuals who had an acute hospitalization in 2013, were aged 65 years or older, and were continuously enrolled in Medicare Part A and B during the 12 months prior to their index hospitalization. We excluded individuals who were enrolled in Medicare Advantage plans, had end-stage kidney disease, or were hospitalized at federal, nonacute care, or critical access hospitals.

### Outcomes, Independent Variables, and Covariates

The outcome of interest was IHT between acute care facilities. As has been done in prior research using these data,^2,4^ a patient was considered to have undergone IHT if the patient had corresponding transfer out and transfer in claims at different hospitals, had a transfer out claim and was admitted to another hospital within 1 day following this claim, or had a transfer in claim and had been discharged from another hospital within 1 day prior to this claim. As in prior studies,^2,4^ we further excluded patients with corresponding transfer out and in claims to the same hospital, patients cared for at outlier hospitals with transfer out rates greater than 35% or transfer in rates of 100%, and patients with more than 1 instance of IHT in the same hospitalization claim.

The primary independent variable of interest was race/ethnicity, as categorized in this data set by the Research Triangle Institute race code (for White, Black, Hispanic, or Other), which uses administrative data rather than self-report.^19^ We use the term Hispanic throughout the manuscript because this is how this ethnicity is reported by CMS.^19^ We focused a priori on Black and Hispanic patients because these groups are traditionally underserved in the US health care system. We use the term disparity generically to refer to differences in health care metrics between population subgroups.^20^

Patient-level and hospital-level covariates were chosen a priori using prior registry-based studies^1,2,3^ exploring variables associated with IHT. Patient-level characteristics were derived from the CMS data set. These included age, sex, Medicaid coinsurance status, median household income by zip code, diagnosis-related group in quartiles as has been done in prior research on this topic,^2^ hierarchical condition category (HCC) score (a measure of comorbidity),^21^ number of admissions in the previous year (ie, 2012), year quarter of admission, and length of stay (LOS) at index hospital prior to transfer. We hypothesized a priori that presence of Medicaid coinsurance and median household income by zip code may be potential mediators of the association between race/ethnicity and IHT, and we accounted for this in our analysis, as described in a later section.

Hospital-level characteristics were derived from the AHA data set. We included hospital size, geographic region (ie, Northeast, Midwest, South, and West), rural-urban commuting area level (ie, metropolitan, nonmetropolitan micropolitan, nonmetropolitan small town, and rural), ownership type (ie, for profit, not for profit, and public), teaching status (ie, major, defined as members of the Council of Teaching Hospitals [COTH]; minor, defined as non-COTH members with a medical school affiliation; and nonteaching), case mix index for all patients hospitalized, and presence of specialty services, including certified trauma center, medical intensive care unit, cardiac intensive care unit, cardiac surgical service, and adult interventional cardiac catheterization, along with a composite score of other available specialty services.^2^

### Statistical Analysis

We used descriptive statistics to define the distribution of variables within the study group. Rao-Scott χ^2^ testing was used to compare categorical variables by race/ethnicity, while analysis of variance testing was used to compare continuous variables; Kruskal-Wallis testing was used to compare LOS.

For our primary analysis, we created a series of staged multivariable logistic regression models to estimate the association between race/ethnicity and IHT. To do so, we incorporated hospital fixed effects and used generalized estimating equations with an independent correlation structure and robust variance estimators to account for patient clustering within hospitals. First, we used a univariable logistic regression model to estimate the unadjusted odds of IHT by race/ethnicity. Second, we used multivariable logistic regression, adjusting for patient characteristics but excluding potential mediator variables of Medicaid co-insurance and median income by zip code. Third, we added hospital fixed effects to the model. Finally, we included potential mediators to the model. In this final logistic regression model, we noted the independent association of other patient characteristics and IHT.

Using our final fully adjusted regression model, we performed several secondary analyses, including stratified analysis by disease and by hospital teaching status, given that most patients who undergo IHT are first evaluated at nonteaching hospitals.^2^ Because Black and Hispanic patients might receive different treatment at hospitals that predominantly serve these groups of patients,^22^ we also performed stratified analyses among patients hospitalized within hospitals in the top decile of Black and Hispanic patient proportion (based on discharge volume), hereafter referred to as Black-serving and Hispanic-serving hospitals.

Analyses were performed using SAS statistical software version 9.4 (SAS Institute). There were no missing data for race/ethnicity, and 10% or less of data was missing for most covariates (Table 1); among 899 557 patients in the study, data for the rural-urban commuting area variable was missing for 184 853 individuals (20.5%), so we created an indicator variable category of missing for this category when included in regression models. For the primary analysis, we considered hypothesis testing statistically significant at a 2-sided *P* value of <.05. For subgroup analysis, we used a Bonferroni correction to account for multiple comparisons; given that we performed 18 subgroup analyses, the adjusted threshold for significance was .05 divided by 18, or .0028. We also report 99.72% CIs for subgroup analyses to account for this.

**Table 1.** Patient and Hospital Characteristics by Race/Ethnicity

Characteristic^a^	No. (%)			*P* value^b^
	White (n = 734 958)	Black (n = 84 544)	Hispanic (n = 47 588)	
Transferred	20 171 (2.7)	1913 (2.3)	1062 (2.2)	<.001
Length of stay prior to transfer, median (IQR), d	2 (1-4)	2 (1-5)	2 (1-5)	<.001
Disease				
AMI	147 875 (20.1)	12 752 (15.1)	8330 (17.5)	<.001
Stroke	171 416 (23.3)	22 718 (26.9)	9851 (20.7)	<.001
Respiratory disease	69 759 (9.5)	6794 (8.0)	4115 (8.7)	<.001
Sepsis	345 908 (47.1)	42 280 (50.0)	25 292 (53.2)	<.001
Patient-level characteristic				
Sex				
Men	344 850 (46.9)	35 622 (42.1)	22 013 (46.3)	<.001
Women	390 108 (53.1)	48 922 (57.9)	25 575 (53.7)	
Age, y				
65-74	210 634 (28.7)	31 195 (36.9)	15 491 (32.6)	<.001
75-84	265 820 (36.2)	29 892 (35.4)	17 987 (37.8)	
≥85	258 504 (35.2)	23 457 (27.8)	14 110 (29.7)	
Medicaid coinsurance	131 206 (17.9)	39 829 (47.1)	29 541 (62.1)	<.001
Median income by zip code quartile^c^				
1st (lowest)	162 912 (22.6)	39 839 (48.7)	17 086 (36.9)	<.001
2nd	186 325 (25.8)	17 668 (21.6)	10 477 (22.6)	
3rd	187 292 (25.9)	14 100 (17.2)	10 956 (23.7)	
4th	185 394 (25.7)	10 267 (12.5)	7753 (16.8)	
DRG weight quartile				
1st	42 389 (5.8)	7031 (8.3)	2006 (4.2)	<.001
2nd	201 952 (27.5)	22 793 (27.0)	12 716 (26.7)	
3rd	331 996 (45.2)	37 038 (43.8)	21 886 (46.0)	
4th	158 621 (21.6)	17 682 (20.9)	10 980 (23.1)	
HCC score summary, mean (SD)	3.31 (1.82)	3.99 (2.23)	3.77 (2.1)	<.001
No. of admissions, prior year (2012)				
0	565 260 (76.9)	63 274 (74.9	38 925 (81.8)	<.001
1	99 455 (13.5)	11 422 (13.5)	4944 (10.4)	
2-3	54 961 (7.5)	7213 (8.5)	2828 (5.9)	
≥4	15 282 (2.1)	2635 (3.1)	891 (1.9)	
Season of admission				
Quarter 1 (January-March)	203 860 (26.7)	22 910 (26.9)	12 824 (27.0)	.002
Quarter 2 (April-June)	186 349 (24.5)	21 164 (24.8)	11 578 (24.3)	
Quarter 3 (July-September)	180 969 (23.7)	20 311 (23.8)	11 300 (23.8)	
Quarter 4 (October-December)	191 073 (25.1)	20 948 (24.6)	11 886 (25.0)	
Index hospital-level characteristic				
No. of beds				
Small (<99)	65 122 (8.9)	4573 (5.4)	2688 (5.7)	<.001
Medium (100-399)	431 625 (58.7)	42 896 (50.7)	27 473 (57.7)	
Large (>400)	238 211 (32.4)	37 075 (43.9)	17 427 (36.6)	
Geographic region				
Northeast	150 848 (20.5)	12 639 (15.0)	7522 (15.8)	<.001
Midwest	181 282 (24.7)	17 303 (20.5)	3775 (7.9)	
South	287 804 (39.2)	48 039 (56.8)	19 305 (40.6)	
West	115 024 (15.7)	6563 (7.8)	16 986 (35.7)	
Ownership				
For profit	96 139 (13.1)	13 834 (16.4)	13 318 (28.0)	<.001
Not-for profit	564 659 (76.8)	59 168 (70.0)	29 307 (61.6)	
Public	74 160 (10.1)	11 542 (13.7)	4963 (10.4)	
Teaching status				
Major	115 833 (15.8)	21 357 (25.3)	7260 (15.3)	<.001
Minor	256 294 (34.9)	28 723 (34.0)	15 572 (32.7)	
Nonteaching	362 831 (49.4)	34 464 (40.8)	24 756 (52.0)	
Rural-urban commuting area–level characteristic				
Metropolitan	357 132 (48.6)	40 771 (48.2)	16 981 (35.7)	<.001
Nonmetro				
Micropolitan	61 744 (8.4)	4817 (5.7)	1599 (3.4)	
Small town	14 503 (12.0)	1292 (1.5)	599 (1.3)	
Rural area	163 144 (22.2)	15 486 (18.3)	13 864 (29.1)	
Missing	138 435 (18.8)	22 178 (26.2)	14 545 (30.6)	
Presence of a certified trauma center^d^	362 812 (55.1)	42 255 (55.5)	23 318 (57.6)	<.001
Presence of intensive care unit				
Medical^d^	643 572 (97.7)	74 980 (98.5)	39 347 (97.1)	<.001
Cardiac^d^	434 038 (65.9)	54 903 (72.2)	26 471 (65.3)	<.001
Presence of cardiac surgical services^d^	442 787 (67.2)	52 040 (68.4)	29 271 (72.3)	<.001
Presence of adult interventional cardiac catheterization^d^	539 230 (81.8)	61 941 (81.4)	33 742 (83.3)	<.001
Composite score of other hospital specialty services, mean (SD)	29.4 (13.3)	30.7 (14.1)	27.2 (14.5)	<.001
Case mix index, mean (SD)	1.59 (0.24)	1.62 (0.27)	1.61 (0.22)	<.001

Abbreviations: AMI, acute myocardial infarction; DRG, diagnosis related group; HCC, hierarchical condition category; IQR, interquartile range.

^a^ Columns may not add to 100% owing to missing data.

^b^ *P* value reported from Rao-Scott χ^2^ testing for categorical variables and analysis of variance testing for continuous variables, except length of stay, which was compared using Kruskal-Wallis testing.

^c^ Data missing for 13 035 White patients (1.8%), 2670 Black patients (3.2%), and 1316 Hispanic patients (2.8%).

^d^ Data missing for 75 999 White patients (10.3%), 8444 Black patients (10.0%), and 7078 Hispanic patients (14.9%).

## Results

Among 899 557 patients, 734 958 patients were White (81.7%), 84 544 patients were Black (9.4%), and 47 588 patients were Hispanic (5.3%); there were 418 683 men (46.5%), and 306 215 patients (34.0%) were 85 years old or older. The mean (SD) age was 76.8 (7.5) years. In the study group, the primary diagnosis was AMI for 174 385 patients (19.4%), stroke for 211 432 patients (23.5%), respiratory disease for 83 865 patients (9.3%), and sepsis for 429 875 patients (47.8%).

In the entire population, 23 952 patients (2.7%) underwent IHT. This included 20 171 White patients (2.7%), 1913 Black patients (2.3%), and 1062 Hispanic patients (2.2%) (Table 1). The median (interquartile range) and mean (SD) LOS were 2 (1-4) days and 3.3 (6.7) days for White patients, 2 (1-5) days and 4.3 (6.8) days for Black patients, and 2 (1-5) days and 3.7 (5.1) days for Hispanic patients (*P* < .001). There were several differences in patient and hospital characteristics by race and ethnicity worth noting. Individuals with Medicaid coinsurance included 39 829 Black patients (47.1%) and 29 541 Hispanic patients (62.1%), compared with 131 206 White patients (17.9%). There were 39 839 Black patients (48.7%) and 17 086 Hispanic patients (36.9%) in the lowest quartile of median income by zip code, compared with 162 912 White patients (22.6%). Black and Hispanic patients also had higher mean (SD) HCC scores (3.99 [2.23] and 3.77 [2.1], respectively) compared with White patients (3.31 [1.82]). A higher proportion of Hispanic patients were hospitalized in the West (16 986 patients [35.7%]) compared with White patients (115 024 patients [15.7%]).

Compared with White patients, the unadjusted odds of undergoing IHT were lower for Black patients (odds ratio [OR], 0.82; 95% CI, 0.76-0.89; *P* < .001) and Hispanic patients (OR, 0.81; 95% CI, 0.72-0.91; P < .001) (Table 2). After adjusting for patient clinical characteristics (but excluding potential mediators), the odds of transfer were lower for Black patients (adjusted OR [aOR], 0.79; 95% CI, 0.76-0.82; *P* < .001) and Hispanic patients (aOR, 0.90; 95% CI, 0.86-0.95; *P* < .001). After incorporating hospital fixed effects, we found that Black patients had persistently lower odds of transfer (aOR, 0.78; 95% CI, 0.73-0.82; *P* < .001), while the estimate was no longer significant for Hispanic patients (aOR, 0.97; 95% CI, 0.90-1.05). Finally, after incorporation of potential mediator variables, Black patients continued to have lower odds of transfer (aOR 0.87; 95% CI, 0.81-0.92; *P* < .001) while Hispanic patients had higher odds of transfer (aOR, 1.14; 95% CI, 1.05-1.24; *P* = .002). This change in estimate in the final model appeared to be primarily associated with differences in Medicaid coinsurance status. In the final model, patients with Medicaid coinsurance were significantly less likely to have undergone IHT compared with those without Medicaid coinsurance (aOR, 0.62; 95% CI, 0.60-0.64; *P* < .001) (eTable 1 in the Supplement).

**Table 2.** Crude and Adjusted Odds of Transfer for Black and Hispanic Patients Compared With White Patients

Race/ethnicity^a^	Crude		Patient-level adjustment					
			With exclusions^b^		With exclusions and fixed effects^c^		All variables^d^	
	OR (95% CI)	*P* value	aOR (95% CI)	*P* value	aOR (95% CI)	*P* value	aOR (95% CI)	*P* value
Black	0.82 (0.76-0.89)	<.001	0.79 (0.76-0.82)	<.001	0.78 (0.73-0.82)	<.001	0.87 (0.81-0.92)	<.001
Hispanic	0.81 (0.72-0.91)	<.001	0.90 (0.86-0.95)	<.001	0.97 (0.90-1.05)	.44	1.14 (1.05-1.24)	.002

Abbreviations: aOR, adjusted odds ratio; OR, odds ratio.

^a^ Reference category is White.

^b^ Excluding Medicaid coinsurance and median income by zip code. Patient-level characteristics include age, sex, hierarchical condition category score, diagnosis related group weight, No. of admissions in 2012, and admission season.

^c^ Excluding Medicaid coinsurance and median income by zip code, with index hospital fixed effects. Hospital-level characteristics include No. of beds; geographic region; ownership type; teaching status; urban location; case mix index; presence of trauma center, medical intensive care unit, cardiac intensive care unit, cardiac surgical service, and adult interventional cardiac catheterization; and composite score of other hospital specialty services.

^d^ Including all patient-level adjustment and index hospital fixed effects.

### Secondary Analysis

In stratified analyses of the final regression model by diagnosis, we found that, among patients with AMI, Black patients had a statistically significant lower adjusted odds of transfer compared with White patients (aOR, 0.73; 99.72% CI, 0.64-0.85; *P* < .001) (Table 3). Upon stratification by hospital teaching status, we found that Black patients had significantly lower adjusted odds of transfer compared with White patients if hospitalized at nonteaching hospitals (aOR, 0.85; 99.72% CI, 0.76-0.94; *P* < .001) or major teaching hospitals (aOR, 0.73; 99.72% CI, 0.55-0.96; *P* = .002) while there was no statistically significant difference for Hispanic patients.

**Table 3.** Adjusted Odds of Transfer for Black and Hispanic Patients Stratified by Patient and Hospital Characteristic^a^

Subgroup	Index hospital fixed effects					
	Black			Hispanic		
	No.	aOR (99.72% CI)^b^	*P* value	No.	aOR (99.72% CI)^b^	*P* value
Patient diagnosis						
AMI	12 752	0.73 (0.64-0.85)	<.001	8330	1.11 (0.93-1.33)	.11
Stroke	22 718	0.87 (0.72-1.04)	.03	9851	1.08 (0.82-1.42)	.47
Respiratory disease	6794	0.68 (0.41-1.12)	.04	4115	1.32 (0.65-2.68)	.29
Sepsis	42 280	0.98 (0.85-1.13)	.72	25 292	1.22 (0.99-1.49)	.009
Hospital teaching status						
Major	21 357	0.73 (0.55-0.96)	.002	7260	0.81 (0.52-1.26)	.20
Minor	28 723	0.94 (0.81-1.10)	.30	15 572	1.20 (0.98-1.47)	.03
Nonteaching	34 464	0.85 (0.76-0.94)	<.001	24 756	1.15 (1.00-1.33)	.006
Hospital minority-serving status^c^						
Minority serving	38 068	0.87 (0.75-1.01)	.01	22 842	1.16 (0.93-1.45)	.08
Not minority serving	47 476	0.85 (0.77-0.94)	<.001	24 746	1.12 (0.98-1.28)	.03

Abbreviations: AMI, acute myocardial infarction; aOR, adjusted odds ratio.

^a^ Reference category is White.

^b^ 99.72% CIs reported to incorporate multiple comparisons and Bonferroni adjustment.

^c^ Minority-serving hospitals include those hospitals in the top 10% of volume of discharges for Black and Hispanic patients, respectively.

Among all patients, 38 068 Black patients (44.5%) and 22 842 Hispanic patients (48.0%) were discharged from Black-serving and Hispanic-serving hospitals, respectively. In stratified analyses, Black patients hospitalized at non-Black–serving hospitals had persistently significantly lower adjusted odds of IHT compared with White patients at these hospitals (aOR, 0.85; 99.72% CI, 0.77-0.94; *P* < .001) (Table 3). There was no statistically significant difference in adjusted odds of transfer for Hispanic patients compared with White patients initially hospitalized at Hispanic-serving or non-Hispanic–serving hospitals.

## Discussion

Using a nationally representative cohort of Medicare patients with common medical diagnoses that have been associated with a mortality benefit from IHT^4^ in this cross-sectional study, we found that Black patients had persistently lower odds of IHT after adjusting for patient and hospital characteristics, while Hispanic patients had significantly greater odds of IHT. These findings largely persisted in analyses stratified by diagnosis. We also found that at nonteaching hospitals, which are more likely to transfer patients compared with teaching hospitals,^2^ and at non-Black–serving hospitals, Black patients had statistically significantly lower adjusted odds of transfer. These findings suggest differential IHT practices based on race/ethnicity.

Our findings add to prior studies^1,2,3,13^ exploring variables associated with IHT whose results that suggest Black patients are less likely to be transferred for a variety of diseases processes, although this has been minimally explored for specific disease entities. Data from a 2018 study^16^ suggest that, after accounting for differences in patient and hospital characteristics, there are racial disparities in interhospital ICU transfer for Black patients with sepsis requiring mechanical ventilation. Similar disparities have been found in rates and timing of transfer for Black patients presenting with AMI to hospitals with revascularization capabilities^5,14,15^ and for Black and Hispanic patients presenting with ischemic stroke.^23^

Our findings suggest that there are several notable associations between race/ethnicity and other included variables that are also associated with odds of IHT and may partially, but not completely, explain our observed results. We found that Black patients had more comorbidities (as measured by HCC score^21^), and HCC score was associated with higher rates of IHT (eTable 1 in the Supplement). After accounting for this confounder and other clinical factors in our adjusted analyses, we found that Black race was associated with lower adjusted odds of IHT. We also found that Black patients had greater rates of Medicaid coinsurance, which was associated with lower odds of IHT (eTable 1 in the Supplement). Thus, adjustment for this mediator moved the aOR toward the null, though the negative association between Black race and IHT persisted.

There are several possible explanations for why we found a persistently lower likelihood of transfer among Black patients. First, our findings may be in part or wholly due to the presence of unmeasured confounders. However, we included a comprehensive list of patient and hospital characteristics in our adjusted analyses; thus, unmeasured confounders are unlikely to fully explain our observed results. Second, Black patients may be less willing to request transfer because of lack of familiarity with the process, greater comfort receiving care at familiar hospitals,^24^ mistrust of the health care system,^25^ or other reasons. We know that some hospital transfers occur at the patient’s request rather than at the clinician’s suggestion.^6^ Relatedly, it is also possible that White patients and those with higher socioeconomic status might feel more empowered to request a transfer. Third, the observed findings may be in part due to differences in the propensity for Black or White patients to present to hospitals that are members of large health care systems. That is, White patients may be more likely than Black patients to present to hospitals that are members of hospital networks, facilitating White patients’ ability to access specialized care at networks hubs.^26^ There may also be associations related to distance between index hospital and nearest referral institutions. However, if present, these differences should also have been controlled for in our hospital fixed effects models. Fourth, it is possible that clinicians harbor implicit bias against Black patients, leading to less recognition of need for transfer or willingness to recommend transfer, supported in part by our findings that Black patients remain at the index hospital longer prior to transfer compared with White patients. There is substantial evidence that implicit bias among clinicians is pervasive in the US medical system and can be associated with differences in treatment recommendations.^27,28,29^ Overall, it is likely that all of these factors, operating individually or in combination for any given patient, are associated with transfer practices and thus the observed disparities in transfer by race/ethnicity.

Our study is among the first to investigate disparities in transfer between Hispanic and White patients, and we surprisingly found that Hispanic patients had higher adjusted odds of transfer compared with White patients in our fully adjusted models. These results differ from those of prior studies^1,16,30,31,32^ that found that Hispanic patients, like Black patients, are less likely to undergo transfer compared with White patients.

There are several possible explanations for our observed associations of Hispanic race/ethnicity and adjusted odds of IHT. First, it is possible that Hispanic patients indeed receive superior access to specialized care for the included medical conditions via IHT. Second, unmeasured confounding and incomplete adjustment for clinical factors may have contributed to our findings, as is suggested by the changing directionality observed with sequential addition of variables in our multivariate modeling. Similar to what we observed with Black patients, this suggests that there is a complex dynamic in operation among Hispanic patients, patient and hospital characteristics, and IHT. These characteristics include comorbidities, Medicaid coinsurance status, and notably geographic location, given that Hispanic patients were more likely to be admitted to hospitals in the West, which had the lowest odds of IHT (eTable 2 in the Supplement). Second, origin hospitals may assume that Hispanic patients are uninsured or underinsured,^10,33^ thus anticipating lower reimbursement rates for services provided and increasing the likelihood that the institution will transfer these patients. There is little evidence to suggest that hospitals receive lower reimbursement rates for patients who are dual-eligible for Medicare and Medicaid,^34^ and in this study, we found Medicaid coinsurance to be associated with lower rates of transfer. However, results from other studies^35,36,37,38,39^ have suggested that underinsured patients are more likely to be transferred. Third, inaccuracies in coding Hispanic patients may contribute to some of these observations, as further discussed in Limitations. Fourth, it may be a combination of the above factors, in addition to other elusive reasons, that lead to these observed findings, necessitating further dedicated evaluation.

### Limitations

This study has several limitations. First, as with any administrative data set, there are potential inaccuracies in coding. Specifically, there are known inaccuracies in coding for Hispanic ethnicity in Medicare data.^19,40,41^ A study comparing 2010 Medicare race/ethnicity data with self-reported survey data found that only one-third of patients were correctly identified as Hispanic.^40^ However, we have no reason to suspect differential misclassification of race by transfer status; therefore, we do not expect systematic bias, only bias toward the null. Second, this data set does not capture indication for transfer. We assume that the majority of patients undergo IHT to receive more specialized care that is unavailable at their hospital of origin; however, in some instances transfer may be initiated owing to provider or patient preference even if the origin hospital is capable of treating the primary disease process.^6,8^ Additional investigation is warranted into how often this may be the case and how race/ethnicity may be associated with preference-related transfers. Third, we did not cluster by clinician and were therefore unable to account for different transfer practices by physicians within a given hospital. Forth, we were unable to capture clinician-patient language concordance, and it may have been useful to account for this when exploring IHT practices among the Hispanic population. Fifth, we could not adjust for availability of beds at receiving hospitals at the time of transfer request in communities with higher numbers of minority patients, and bed availability may have influenced the hospital’s ability to accept a patient for transfer. Sixth, we were unable to account for unmeasured potentially confounding variables that may influence the association between race/ethnicity and IHT.

## Conclusions

This cross-sectional study found differences in IHT by race/ethnicity among patients with common medical conditions for which transfer has been associated with a mortality benefit. We found that, after accounting for patient and hospital characteristics, Black patients were consistently significantly less likely compared with White patients to undergo IHT across various hospital settings. Conversely, Hispanic patients overall were more likely to be transferred compared with White patients. While our analysis cannot fully explain why such variations were found, given the known prevalence of systemic biases by race/ethnicity in the US health care system, further investigation is warranted to determine the underlying reasons for these differences and what strategies may be employed to mitigate disparate treatment by race/ethnicity.

## Notes

Supplement. **eTable 1.** Adjusted Odds of Transfer for All Diagnoses for Patient-Level Variables After Adjustment, Using Hospital Fixed Effects **eTable 2.** Adjusted Odds of Transfer for All Diagnoses, Incorporating Hospital Characteristics
