# Supplementary material for: Racial/Ethnic Disparities in Interhospital Transfer for Conditions With a Mortality Benefit to Transfer Among Patients With Medicare
Source: JAMA Netw Open. 2021 Mar 26;4(3):e213474. doi: 10.1001/jamanetworkopen.2021.3474 (PMC7998076; doi:10.1001/jamanetworkopen.2021.3474)
Supplement: Supplement. — eTable 1. Adjusted Odds of Transfer for All Diagnoses for Patient-Level Variables After Adjustment, Using Hospital Fixed Effects eTable 2. Adjusted Odds of Transfer for All Diagnoses, Incorporating Hospital Characteristics [file jamanetwopen-e213474-s001.pdf]

Supplemental Online Content

Shannon EM, Zheng J, Orav EJ, Schnipper JL, Mueller SK. Racial/ethnic disparities in interhospital transfer for conditions with a mortality benefit to transfer among patients with Medicare. *JAMA Netw Open*. 2021;4(3):e213474. doi:10.1001/jamanetworkopen.2021.3474

eTable 1. Adjusted Odds of Transfer for All Diagnoses for Patient-Level Variables After Adjustment, Using Hospital Fixed Effects
eTable 2. Adjusted Odds of Transfer for All Diagnoses, Incorporating Hospital Characteristics

This supplemental material has been provided by the authors to give readers additional information about their work.

© 2021 Shannon EM et al. *JAMA Network Open.*

eTable 1. Adjusted Odds of Transfer for All Diagnoses for Patient-Level Variables After Adjustment, Using Hospital Fixed Effects

Characteristic	aOR (95% CI)	p-value
Race/ethnicity		
White	Ref	
Black	0.87 (0.81-0.92)	<0.001
Latinx	1.14 (1.05-1.24)	0.002
Other	1.26 (1.15-1.37)	<0.001
Male sex	1.14 (1.11-1.17)	<0.001
Age		
65-74	4.06 (3.89-4.23)	<0.001
75-84	2.75 (2.64-2.86)	<0.001
85+	Ref	
Medicaid Co-Insurance	0.62 (0.60-0.64)	<0.001
Median income by zip code quartile		
1st	0.95 (0.90-1.01)	0.13
2nd	0.95 (0.90-1.00)	0.06
3rd	0.96 (0.91-1.01)	0.11
4th	Ref	
DRG weight quartile		
1st	6.22 (5.86-6.60)	<0.001
2nd	2.32 (2.20-2.45)	<0.001
3rd	1.82 (1.74-1.92)	<0.001
4th	Ref	
HCC Score Summary	1.32 (1.31-1.33)	<0.001
Number of admissions, 2012		
0	2.00 (1.79-2.23)	<0.001
1	1.56 (1.39-1.75)	<0.001
2-3	1.21 (1.17-1.46)	<0.001
4+	Ref	
Season of admission		

© 2021 Shannon EM et al. *JAMA Network Open.*

Q1 (Jan-Mar)	1.12 (1.08-1.17)	<0.001
Q2 (Apr-Jun)	1.15 (1.10-1.20)	<0.001
Q3 (Jul-Sept)	1.20 (1.15-1.25)	<0.001
Q4 (Oct-Dec)	Ref	

Abbreviations: AMI, acute myocardial infarction; CMI, case mix index; DRG, diagnosis related groups; HCC, Hierarchical Condition Category

© 2021 Shannon EM et al. *JAMA Network Open.*

eTable 2. Adjusted Odds of Transfer for All Diagnoses, Incorporating Hospital Characteristics

Characteristic	aOR (95% CI)	p-value
**Patient-level**		
Race/ethnicity		
White	Ref	
Black	0.81 (0.76-0.87)	<0.001
Latinx	1.04 (0.95-1.13)	0.39
Other	1.25 (1.16-1.36)	<0.001
Male sex	1.13 (1.10-1.17)	<0.001
Age		
65-74	3.88 (3.70-4.06)	<0.001
75-84	2.67 (2.55-2.79)	<0.001
85+	Ref	
Medicaid Co-Insurance	0.63 (0.61-0.66)	<0.001
Median income by zip code quartile		
1st	1.07 (0.99-1.15)	0.08
2nd	1.00 (0.94-1.04)	>0.99
3rd	0.95 (0.90-1.00)	0.05
4th	Ref	
DRG weight quartile		
1st	6.23 (5.82-6.65)	<0.001
2nd	2.54 (2.40-2.70)	<0.001
3rd	1.98 (1.87-2.09)	<0.001
4th	Ref	
HCC Score Summary	1.31 (1.29-1.32)	<0.001
Number of admissions, 2012		
0	1.91 (1.70-2.14)	<0.001
1	1.53 (1.35-1.72)	<0.001
2-3	1.29 (1.13-1.46)	0.0001
4+	Ref	

© 2021 Shannon EM et al. *JAMA Network Open.*

Season of admission		
Q1 (Jan-Mar)	1.12 (1.08-1.17)	<0.001
Q2 (Apr-Jun)	1.15 (1.10-1.20)	<0.001
Q3 (Jul-Sept)	1.19 (1.14-1.24)	<0.001
Q4 (Oct-Dec)	Ref	
**Index Hospital-level**		
Bedsize		
Small (<99 beds)	2.15 (1.74-2.66)	<0.001
Medium (100-399 beds)	1.72 (1.42-2.07)	<0.001
Large (≥400 beds)	Ref	
Hospital Region		
Northeast	1.56 (1.38-1.77)	<0.001
Midwest	1.30 (1.16-1.44)	<0.001
South	1.05 (0.94-1.17)	0.40
West	Ref	
Ownership		
Not-for-profit	Ref	
Public	1.06 (0.96-1.18)	0.26
For profit	1.19 (1.06-1.34)	0.003
Teaching status		
Minor	Ref	
Non-teaching	1.23 (0.98-1.54)	0.08
Major	1.42 (1.12-1.81)	0.003
RUCA level		
Metropolitan	Ref	
Nonmetro, micropolitan	1.29 (1.18-1.41)	<0.001
Nonmetro, small town	1.33 (1.17-1.150)	<0.001
Rural	1.09 (0.99-1.19)	0.07
Missing	0.95 (0.86-1.05)	0.33
Presence of Medical ICU	1.14 (1.01-1.29)	0.03
Presence of Cardiac ICU	0.85 (0.78-93)	<0.001

© 2021 Shannon EM et al. *JAMA Network Open.*

Presence of Adult Interventional Cardiology	0.67 (0.61-0.73)	<0.001
Presence of Cardiac Surgery	0.25 (0.22-0.30)	<0.001
Presence of Certified Trauma Center	1.10 (1.03-1.18)	0.008
Hospital composite score	0.99 (0.99-1.00)	0.09
Case mix Index	0.33 (0.24-0.43)	<0.001
With Medical ICU		

Abbreviations: AMI, acute myocardial infarction; CMI, case mix index; DRG, diagnosis related groups; HCC, Hierarchical Condition Category

© 2021 Shannon EM et al. *JAMA Network Open.*
